# Fascial dehiscence after radical cystectomy: Is abdominal exploration mandatory?

**DOI:** 10.1186/s12894-022-01095-4

**Published:** 2022-09-03

**Authors:** Paz Lotan, Shayel Bercovich, Daniel Keidar, Kamil Malshy, Ziv Savin, Rennen Haramaty, Jonathan Gal, Jonathan Modai, Dan Leibovici, Roy Mano, Barak Rosenzweig, Azik Hoffman, Miki Haifler, Jack Baniel, Shay Golan

**Affiliations:** 1 Department of Urology,, Rabin Medical Center, 49372 Petach Tikva, Israel; 2 Sackler School of Medicine, Tel Aviv University, Tel Aviv, Israel; 3 Department of Urology, Rambam Health Care Campus, Haifa, Israel; 4 Rappaport Faculty of Medicine, Technion – Israel Institute of Technology, Haifa, Israel; 5 Department of Urology, Tel-Aviv Sourasky Medical Center, Tel Aviv, Israel; 6 Department of Urology, The Chaim Sheba Medical Center at Tel Hashomer, Ramat-Gan, Israel; 7 Department of Urology,, Shamir Medical Center at Assaf Harrofeh, Tzrifin, Israel; 8 Department of Urology, Kaplan Medical Center, Rehovot, Israel

**Keywords:** Fascia, Dehiscence, Bladder cancer, Cystectomy, Surgery, Laparotomy

## Abstract

**Background:**

Fascial dehiscence after radical cystectomy may have serious clinical implications. To optimize its management, we sought to describe accompanying intraabdominal findings of post-cystectomy dehiscence repair and determine whether a thorough intraabdominal exploration during its operation is mandatory.

**Methods:**

We retrospectively reviewed a multi-institutional cohort of patients who underwent open radical cystectomy between 2005 and 2020. Patients who underwent exploratory surgery due to fascial dehiscence within 30 days post-cystectomy were included in the analysis. Data collected included demographic characteristics, the clinical presentation of dehiscence, associated laboratory findings, imaging results, surgical parameters, operative findings, and clinical implications. Potential predictors of accompanying intraabdominal complications were investigated.

**Results:**

Of 1301 consecutive patients that underwent cystectomy, 27 (2%) had dehiscence repair during a median of 7 days post-surgery. Seven patients (26%) had accompanying intraabdominal pathologies, including urine leaks, a fecal leak, and an internal hernia in 5 (19%), 1 (4%), and 1 (4%) patients, respectively. Accompanying intraabdominal findings were associated with longer hospital stay [20 (IQR 17, 23) vs. 41 (IQR 29, 47) days, P = 0.03] and later dehiscence identification (postoperative day 7 [IQR 5, 9] vs. 10 [IQR 6, 15], P = 0.03). However, the rate of post-exploration complications was similar in both groups. A history of ischemic heart disease was the only predictor for accompanying intraabdominal pathologies (67% vs. 24%; P = 0.02).

**Conclusions:**

A substantial proportion of patients undergoing post-cystectomy fascial dehiscence repair may have unrecognized accompanying surgical complications without prior clinical suspicion. While cardiovascular disease is a risk factor for accompanying findings, meticulous abdominal inspection is imperative in all patients during dehiscence repair. Identification and repair during the surgical intervention may prevent further adverse, possibly life-threatening consequences with minimal risk for iatrogenic injury.

## Background

Bladder cancer is the fourth most common cancer in men and the eleventh most common in women, with an estimated 549,000 new cases and 200,000 deaths annually [1]. Muscle invasive disease represents more than a fifth of newly diagnosed bladder cancers [2]. The standard of care for localized disease is radical cystectomy (RC) with bilateral pelvic lymph node dissection [3].

Despite its wide application and perioperative advancements, RC is associated with a ~ 60% complication rate [3–6]. Fascial dehiscence (FD) occurs in up to 9% of cases and is often accompanied by increased morbidity, including wound infection, prolonged hospitalization, incisional hernia, and reoperation. It is also associated with a negative body image, decreased quality of life, and increased care costs [5–11].

While risk factors for FD after RC have been well studied [12, 13], there is little consensus on its management once it has occurred. Some minor fascial disruptions may be treated conservatively, but the majority are usually managed surgically [14]. Such reoperation may, or may not, include complete abdominal exploration to rule out any accompanying pathology that may have predisposed the patient to dehiscence. Although RC patients are at increased risk for complications, such as foci or anastomotic leak, thorough abdominal exploration may increase the risk for iatrogenic injury and prolong operative time.

To optimize FD management, we set out to describe intraabdominal findings and surgical outcomes of post-cystectomy dehiscence repair and determine whether a thorough intraabdominal exploration during its operation is mandatory.

## Methods

We retrospectively reviewed the medical records of a multi-institutional cohort of patients in six academic centers who had open RC between January 2005 and April 2020. We identified those who underwent FD repair within 30 days post-RC. Patients were included in the analysis if the indication for surgical exploration was solely FD, while those with other clinical indications for intervention were excluded. All patients had thorough, systematic abdominal explorations inspecting all anastomoses, surgical sites, and intestinal integrity.

Data collected included demographic characteristics, the clinical presentation of FD, associated laboratory findings, imaging results, surgical parameters, and operative findings. To better characterize the patients' medical state immediately before FD repair, the Acute Physiology And Chronic Health Evaluation (APACHE) II score was calculated for each patient. The APACHE II score is a well-established classification system for the severity of diseases, including acute abdominal pathologies in surgical patients [15, 16]. Documented operative findings during dehiscence repair included intraabdominal purulent fluid, bowel or urinary leakage, and any intestinal abnormalities. Other surgical parameters included operative length, estimated blood loss, adverse events, and the fascial closure method. Potential predictors of the accompanying intraabdominal pathologies and their clinical consequences were also evaluated.

### Statistical analysis

Categorical variables were summarized by number and percentage, and continuous variables were summarized by mean and standard deviation (SD) or median and interquartile range (IQR). Categorical variables were compared using Fisher’s exact test, and continuous variables were compared using the Mann–Whitney or t-test as appropriate. Statistical analyses were performed using SAS version 9.4 (SAS Institute Inc., Cary, NC, USA) with a two-sided significance level set at p < 0.05.

## Results

Of 1301 consecutive patients who underwent RC between 2005 and 2020, we identified 36 (2.8%) that were diagnosed with post open RC FD and underwent explorative laparotomy. Nine patients had a concordant indication for a surgical intervention (bowel obstruction [n = 4], fecal leak [n = 4], and a urinary leak [n = 1]). The remaining 27 patients (2%) had FD as the only indication for surgical exploration and comprised the study cohort.

The patients' baseline characteristics and RC perioperative parameters are summarized in Tables 1 and 2, respectively. Median age was 75 years (IQR 68, 80) and 26 (96%) were males. The urinary diversion of choice was Ileal conduit in 24 patients (89%) and orthotopic neobladder in 3 (11%). Median RC operative time was 353 min (IQR 275, 414). Continuous nonabsorbable or slowly absorbable monofilament sutures were used for fascial closure.

**Table 1:** Baseline characteristics of patients with fascial dehiscence after radical cystectomy

Characteristic	Total N (%)	Patients with accompanying intra-abdominal complications	Patients without accompanying intra-abdominal complications	*P*
No. of patients (%)	27 (100)	7 (26)	20 (74)	
Gender				
Male	26 (96)			
Female	1 (4)			
Age (years)	75 (68, 80)	78 (72, 81)	74 (65, 80)	0.19
Body mass index kg/m^2^	29 (26, 31)	30 (27, 32)	29 (26, 30)	0.65
Smoking past/present	21 (78)	6 (86)	15 (75)	0.56
Diabetes melitus	9 (33)	4 (57)	5 (25)	0.12
Peripheral vascular disease	4 (15)	1 (14)	3 (15)	1
Ischemic heart disease	9 (33)	5 (71)	4 (20)	**0.02**
Cerebrovascular accident	1 (4)	1 (14)	0	0.26
Cardiac arrhythmia	7 (26)	3 (43)	4 (20)	0.23
Steroid use	2 (7)	0	2 (10)	1
Chronic lung disease	4 (15)	0	4 (20)	0.54
Antiplatelet/anticoagulation use	11 (41)	5 (71)	6 (30)	**0.05**
Previous abdominal operation	4 (15)	0	4 (20)	0.55
Neoadjuvant chemotherapy	6 (22)	2 (29)	4 (20)	0.63
Age-adjusted Charlson comorbidity index	6 (5, 8)	7 (6, 10)	6 (5, 8)	0.19

*IQR* interquartile range; Continuous variables are shown as median (IQR) and categorical variables are shown as number and percentage

**Table 2:** Operative and postoperative parameters of radical cystectomy

Characteristic	Total N = 27 (%)	Patients with accompanying intra-abdominal complications N = 7	Patients without accompanying intra-abdominal complications N = 20	*P*
Type of urinary diversion				
Ileal conduit	24 (89)	7 (100)	17 (85)	0.55
Orthotopic neobladder	3 (11)	0	3 (15)	
Operating room time (min)	353 (275, 414)	355 (275, 450)	349 (264, 406)	0.9
Estimated blood loss (ml)	500 (450, 1000)	550 (500, 700)	500 (425, 1000)	0.88
Admitted to the intensive care unit (post-cystectomy)	4 (15)	2 (29)	2 (10)	0.27
Other preioperative complications^†^	19 (70)	5 (71)	14 (70)	1
Respiratory failure	2	1	1	
Cerebrovascular accident	1	0	1	
Prolonged ileus	3	0	3	
Deep venous thrombosis	2	1	1	
Pneumonia	1	1	0	
Cardiac arrhythmia	2	0	2	
Urinary tract infection	12	2 (29)	10 (50)	0.33
Wound infection	16 (59)	5 (71)	11 (55)	0.12
Wound pathogen resistant to preoperative anibiotics	12 (75)	5 (100)	7 (64)	0.25
Post-operative day of dehiscence diagnosis	7 (5, 10)	10 (6, 15)	7 (5, 9)	**0.03**
Pre-dehiscence Albumin g/dL	2.8 (2.5, 3.6)	2.5 (2.2, 3.5)	2.8 (2.6, 3.1)	0.38
Pre-dehiscence Creatinine mg/dL	1.14 (0.9, 1.4)	1.15 (1.1, 1.4)	1.11 (0.85, 1.3)	0.36
Pre-dehiscence Hemoglobin g/dL	10.2 (9.7, 11)	9.9 (9.6, 11)	10.3 (9.9, 11.2)	0.38
Pre-dehiscence Leukocyte count 10^3^/µl	10.7 (8.2, 12.8)	11.7 (8.3, 19)	10.6 (7.3, 12.4)	0.2
Pre-dehiscence C-reactive protein mg/dL	12.4 (9.2, 18.7)	19.8 (11.2, 22.8)	11.6 (9.6, 13.1)	0.2
APACHE II score	10.4 (2.9)	10.1 (1.7)	10.5 (3.3)	0.8

Continuous variables are shown as median (IQR) and categorical variables are shown as number and percentage. APACHE score, shown as mean and standard deviation

*APACHE* Acute Physiology and Chronic Health Evaluation, *IQR* interquartile range, *SD* standard deviation

^†^Might be more than one complication per patient

FD was diagnosed within a median of 7 days (IQR 5, 10) after RC. The clinical presentation was seepage of pink serosanguinous fluid from the wound in all patients. Additionally, accompanying evisceration was diagnosed in 13 (48%) patients.

Table 3 summarizes the perioperative surgical parameters, the findings during FD repair, and the clinical implications.

**Table 3:** Fascial dehiscence: operative and postoperative parameters

Characteristic	Total N = 27	Patients with accompanying intra-abdominal complications N = 7	Patients without accompanying intra-abdominal complications N = 20	*P*
Length of laparotomy (minutes)	60 (49, 98)	60 (60, 115)	64 (47, 89)	0.3
Type of complication^†^				
Anastomotic ureteral dehiscence		4 (15)		
Ileal conduit perforation		1 (4)		
Infected collection/abscess		1 (4)		
Gastrointestinal anastomotic leakage		1 (4)		
Internal hernia		1 (4)		
Primary fascial closure	25 (93)	5 (71)	20 (100)	
Perioperative complications	10 (37)	3 (43)	7 (35)	1
Admitted to ICU (post-laparotomy)	7 (26)	3 (43)	4 (20)	0.33
No. of days in ICU	7 (1.5, 7.5)	8 (4.5, 19.5)	4.5 (1.8, 7)	0.32
Length of hospital stay	23 (17, 29)	41 (29, 47)	20.5 (17, 23.5)	**0.03**
Late complications	5 (19)	1 (14)	4 (20)	1
Perioperative mortality	3 (11)	1 (14)	2 (10)	1
Total mortality during follow-up	13 (48)	2 (29)	11 (55)	0.4

Continuous variables are shown as median (IQR) and categorical variables are shown as number and percentage

*ICU* intensive care unit, *IQR* interquartile range

^†^Might be more than one complication per patient

During the repair, a fascial tear was found in 25 patients (93%) and a suture disruption in 2 (7%). Abdominal exploration was performed in all 27 patients, and accompanying intraabdominal pathologies were found in 7 (26%). These included an ileo-ureteric anastomotic leak (n = 4, 15%), an ileal conduit urinary leakage at the suture lines (n = 1, 4%), a fecal leakage at the ileo-ileal anastomosis (n = 1, 4%), purulent abdominal fluid collection (n = 1, 4%), and internal hernia (n = 1, 4%). Two patients with accompanying intraabdominal pathologies had a contrast CT before surgical exploration to confirm the FD. However, they did not have other abnormal findings except for the FD in the scans. The mean perioperative APACHE II score was similar between patients with or without accompanying intraabdominal pathologies (10.1 ± 1.7 vs. 10.5 ± 3.3, p = 0.8).

The median laparotomy time was 60 min (IQR 48, 98), and primary fascial closure was performed in 25 patients (93%). Two patients (7%) had delayed fascial closure due to the additional findings. The patient who was found to have an internal hernia required resection of a short bowel segment and primary intestinal anastomosis. Moreover, iatrogenic bowel injury occurred in 1 patient (4%) during adhesiolysis. This injury was primarily repaired.

All facial repairs were done with slowly absorbable 0 polydioxanone (PDS) loop (n = 18, 67%), or nonabsorbable monofilament 1 nylon loop (n = 9, 33%). An absorbable prosthetic mesh was used in 3 (11%) patients due to large fascial tears. A continuous single-layer midline suture technique with a suture to wound length ratio of at least 4:1 was used. The sutures were put according to the traditional 1 cm from the fascial edge and 1 cm advance.

Post-laparotomy complications within 30 days of the procedure were reported in 10 patients (37%): 3 patients died (1 of sepsis, 1 of pneumonia, and 1 of myocardial infarction). Only the patient that died of sepsis had an accompanying intraabdominal pathology (ileo-ileal anastomotic leak). Other 30-day post-laparotomy complications included febrile urinary tract infection (3 patients, 11%), prolonged ileus (3 patients, 11%), pneumonia (1 patient, 4%), and severe metabolic acidosis (1 patient, 4%). The median total length of hospital stay was 23 days (IQR 17, 29).

When comparing the group of patients with accompanying intraabdominal findings (i.e. bowel or urinary leak, purulent abdominal fluid, or internal hernia) and those without findings, we found that the latter was diagnosed with dehiscence earlier (postoperative day 7 [IQR 5, 9] vs. postoperative day 10 [IQR 6, 15], P = 0.03) and had a shorter length of hospital stay (20 days [IQR 17, 23.5] vs. 41 days [IQR 29, 47], P = 0.03]. The only potential predictor of accompanying intraabdominal pathologies was ischemic heart disease (IHD) which was more common in this subgroup compared to patients with dehiscence only (71% vs. 20%. P = 0.02). We also noticed a trend toward increased blood thinner usage among patients with accompanying findings, with borderline statistical significance (71% vs. 30%, P = 0.05). No other potential clinical, laboratory or radiologic predictors of accompanying intraabdominal pathologies were identified (Table 3).

During a median follow-up of 28 months (IQR 9, 48), 5 patients (19%) developed late complications. Two patients (7%) developed ventral hernia, 2 (7%) ureteral anastomotic stricture, and 1 (4%) entero-cutaneous fistula. The accompanying intraabdominal pathologies found during the FD repair were not associated with these late sequelae.

## Discussion

FD after RC is a dreaded complication requiring improved characterization and management guidance. Our multicenter study shows that a significant percentage of concomitant intraabdominal pathologies were identified only at the time of dehiscence repair. These findings highlight the need for thorough abdominal exploration and prompt diagnosis to prevent additional morbidity.

FD was previously found to occur in up to 9% of RC [5]. However, more contemporary series reported a rate of 3% [7, 13], similar to our results. Identification of risk factors and preventive measures may have contributed to the reduced rate of FD. Potentially modifiable risk factors include patient-related factors, such as preoperative malnutrition, smoking, body mass index ≥ 25, postoperative coughing, and technical errors during wound closure [10, 17]. Despite efforts to control preoperative, intraoperative, and postoperative factors, the majority of patients will require surgical repair once FD occurs [14].

Although the fastest and simplest surgical intervention to repair FD is by primary closure of the fascial edges, FD might be accompanied by intraabdominal pathologies [17]. While these complications may serve as potential predisposing factors for FD, they are not always clinically evident. In our cohort, accompanying complications were identified in a quarter of patients only at the time of fascial repair. Most findings were anastomotic leaks that were amenable to primary repair. Indeed, the surgical complexity of RC is related to the urinary-gastrointestinal anastomoses, usually performed in patients with comorbid conditions that may increase the risk for anastomotic breakdown.

FD was diagnosed and repaired after a median time of 7 days following cystectomy, similar to previous reports, and around the time of the susceptible proliferative and remodeling phases of wound repair [8, 9, 13, 14, 18]. Interestingly, patients with accompanying intraabdominal findings were diagnosed with FD three days later than the rest. While the reason for that is not apparent, it is possible that in patients with intraabdominal pathologies the mechanism and course of FD development differs from that of patients without intraabdominal pathologies.

While abdominal imaging is not routinely performed before FD repair, two of our cohort's seven patients who had accompanying intraabdominal pathologies underwent a contrast CT to support FD diagnosis. However, the urography phase was not included in these CT scans, and the urinary leaks went undiagnosed. Therefore, if the decision to perform a CT is made, the urography phase should be included.

The only clinical factor found to predict the accompanying pathologies was IHD. Atherosclerosis leads to tissue hypoperfusion, decreased oxygenation, and impaired tissue healing. Therefore, it is reasonable that patients with IHD will be at an increased risk for wound complications and anastomotic breakdown, causing urine or fecal leak.

Although hospital stay was longer among patients with accompanying intraabdominal pathologies, the rate of post-exploration complications was similar in these patients compared to patients who only had FD. Identifying these pathologies and their primary repair during the abdominal exploration might have prevented additional adverse outcomes. Nevertheless, it should be mentioned that during the abdominal exploration, iatrogenic injury to the ileo-ileal anastomosis occurred in one patient, which was primarily sutured without further complications.

This study's overall 30-day mortality rate was 11%, higher than the 2–3% reported for general RC series [3, 4]. There was no association between mortality and accompanying intrabdominal pathologies. This high mortality rate probably reflects a significantly increased surgical risk in the FD population. Patients in this study were older and sicker than those in previous studies (median Charlson comorbidity index = 6) [4, 14], two parameters that are associated with post-RC mortality [19]. Similarly, high mortality rates of up to 45% have been reported in patients who experienced FD after colorectal surgeries [9, 10].

Late complications after FD repair are not uncommon. They are associated with additional physical and mental burdens and further increase the cost of care [11, 17]. For example, an incisional hernia carries the risk of subsequent bowel obstruction and the need for additional procedures. In our study, accompanying intraabdominal pathologies did not increase the risk for late complications and did not impact overall survival. It is reasonable to assume that identifying and correcting these pathologies assisted in preventing further sequelae.

Our study is limited by its retrospective design and small sample size. The lack of standardized diagnostic evaluation before the surgical intervention may have impacted our findings. Specifically, some of the surgical findings would have been identified by contrast CT scan with urography phase performed prior to FD repair.

Furthermore, our data was collected from several tertiary referral centers, and the studied event is relatively uncommon, precluding other study types. The lack of a control group might hamper our assumption that the primary correction of accompanying intraabdominal findings prevented further consequences. However, even if some of the complications would have resolved spontaneously, further delay in diagnosing severe complications, such as bowel leak, could have led to life-threatening clinical deterioration. We have also shown that the clinical benefit appears to overcome the potential risk of surgical exploration. There was a single case of iatrogenic injury, amendable for immediate repair. Eventually, long-term morbidity and mortality were not higher in patients with accompanying intraabdominal pathologies.

Our results shed light on the prevalence, management, and outcomes of adverse intraabdominal findings during FD repair post-RC. FD may be the tip of the iceberg, and accompanying complications may remain undetected in a significant proportion of patients. Until now, there was no consensus on whether a complete abdominal exploration is mandatory during post-RC dehiscence repair. Our findings suggest a need for heightened vigilance due to possible subclinical complications associated with FD. Therefore, exploratory laparotomy should be performed while carefully examining all anastomotic sites.

## Conclusions

Clinically unsuspected intraabdominal complications occur in a substantial proportion of patients who undergo FD repair after RC. These mainly include anastomotic leaks that can be identified and safely corrected during the surgical intervention. A history of cardiovascular disease is a risk factor for concordant intraabdominal pathologies; therefore, meticulous abdominal inspection is imperative for prompt diagnosis and repair, especially in this population.

## Data Availability

The datasets generated during and/or analysed during the current study are available from the corresponding author on reasonable request.

## Abbreviations

APACHE: Acute Physiology And Chronic Health Evaluation
FD: Fascial dehiscence
IHD: Ischemic heart disease
IQR: Interquartile range
RC: Radical cystectomy
SD: Standard deviation
